# ApreciseKUre: an approach of Precision Medicine in a Rare Disease

**DOI:** 10.1186/s12911-017-0438-0

**Published:** 2017-04-14

**Authors:** Ottavia Spiga, Vittoria Cicaloni, Andrea Bernini, Andrea Zatkova, Annalisa Santucci

**Affiliations:** 1 Department of Biotechnology, Chemistry and Pharmacy, University of Siena, Siena, Italy; 2 Toscana Life Sciences Foundation, Siena, Italy; 3 Institute for Clinical and Translational Research Biomedical Research Center, Slovak Academy of Sciences Bratislava, Bratislava, Slovakia

**Keywords:** Alkaptonuria, Rare disease, Precision Medicine, Database, Data analysis, Biomarkers

## Abstract

**Background:**

Alkaptonuria (AKU; OMIM:203500) is a classic Mendelian genetic disorder described by Garrod already in 1902. It causes urine to turn black upon exposure to air and also leads to ochronosis as well as early osteoarthritis.

**Main body of the abstract:**

Our objective is the implementation of a Precision Medicine (PM) approach to AKU. We present here a novel ApreciseKUre database facilitating the collection, integration and analysis of patient data in order to create an AKU-dedicated “PM Ecosystem” in which genetic, biochemical and clinical resources can be shared among registered researchers. In order to exploit the ApreciseKUre database, we developed an analytic method based on Pearson’s correlation coefficient and *P* value that generates as refreshable correlation matrix. A complete statistical analysis is obtained by associating every pair of parameters to examine the dependence between multiple variables at the same time.

**Short conclusions:**

Employing this analytic approach, we showed that some clinically used biomarkers are not suitable as prognostic biomarkers in AKU for a more reliable patients’ clinical monitoring. We believe this database could be a good starting point for the creation of a new clinical management tool in AKU, which will lead to the development of a deeper knowledge network on the disease and will advance its treatment. Moreover, our approach can serve as a personalization model paradigm for other inborn errors of metabolism or rare diseases in general.

**Electronic supplementary material:**

The online version of this article (doi:10.1186/s12911-017-0438-0) contains supplementary material, which is available to authorized users.

## Background

Precision medicine (PM) is an emerging approach for disease prevention, diagnosis and treatment that takes into account individual variability in genes, environment, metabolomics, proteomics and lifestyle [1]. The ability to collect, integrate and analyse relevant data streams is the core for developing a “Precision Medicine Ecosystem” in which genetic, biochemical and clinical resources are shared between researchers, clinicians and patients [2]. Computational modelling and database building can be a useful guide to generate an exhaustive and dynamic picture of the individual and to identify potential new biomarkers. Improvements in the knowledge of disease mechanisms and in the biomarkers identification are opening new opportunities to match therapy to patient, and thus leading to a more personalized medicine for maximizing the benefit-to-risk ratio. Ideally, biomarkers identification should be done as early as possible in the workflow of drug discovery in order to stratify dissimilar patient groups [3]. Nowadays PM is used in many areas of health (e.g., cancer), however, this approach was originally developed to manage and analyze data regarding rare diseases.

Alkaptonuria (AKU) (OMIM:203500) is the very first genetic disorder to be described, as reported by Garrod in 1902 [4]. AKU is an autosomal recessive inborn error of metabolism of the amino acids tyrosine due to a deficient activity of the enzyme homogentisate 1,2-dioxygenase (HGD). This leads to the accumulation of homogentisic acid (HGA, 2,5-dihydroxyphenylacetic acid) which is excreted in the urine, giving it an unusually dark colour. The accumulation of HGA in connective tissues is responsible for discoloration of bone (a process called ‘ochronosis’) inducing an early-onset painful osteoarthritis. The oxidation of HGA produces free radical species that are associated with tissue oxidative damage and are thought to cause degeneration by inciting inflammation [5]. In some patients, aortic valve stenosis was reported, as well as kidney and prostate stones. Additionally, AKU also implied a secondary amyloidosis, one of the most severe complications of several chronic rheumatic disorders due to the deposition of amyloid in the synovial membranes of the joint or of the tendon sheats [6]. Usually, at early-stages of the disease patients are given anti-inflammatories or pain killers, while for end-stages joint and heart valve replacement is often required. Since 2012, the DevelopAKUre project is underway, aimed to test nitisinone as a possible treatment for AKU. It is believed that it can prevent or slow down progression of AKU [7].

In order to prevent or minimize the effects of ochronotic arthropathy, a PM approach, tailoring pharmacological, surgical and dietary treatment, would be of great value for AKU patients care. The establishment of a database, which would integrate patient-derived information (quality of life), clinician-derived information (test results, genotypes), and mutational analysis (protein molecular modeling) offers an exhaustive visualization of different informational layers to support clinicians and researchers in PM application to AKU. It is an innovative approach that could not only advance the treatment of AKU, but also to serve as a model for other rare diseases.

## Construction and content

The interactive database for AKU, ApreciseKUre database (http://www.sbl.unisi.it/aprecisekure), integrates data on mutations, patient quality of life and clinical outcomes. It is proposed as a supporting online tool for clinicians to be used in order to practice “PM” in the context of AKU. Data has been primarily collected from *HGD* mutation database (http://hgddatabase.cvtisr.sk/) that includes all *HGD* variants in AKU patients reported so far [8]. Later we have integrated our results from proteomic studies in AKU, information obtained through collaborations with patients’ organizations in Europe (http://www.aimaku.it/, http://www.akussac.sk/, http://www.alcap.fr/, http://www.akusociety.org/, http://www.dsaku.de/), as well as data from SONIA1 clinical study [7]. A coding system is used to anonymize data. The database integrates different types of information regarding genotype, biomarkers, environment, lifestyle, habit, histopathologic, clinical and therapies of patients. In particular, the database schema is divided into 11 tables (see Additional file 1).

**Fig. 1:**
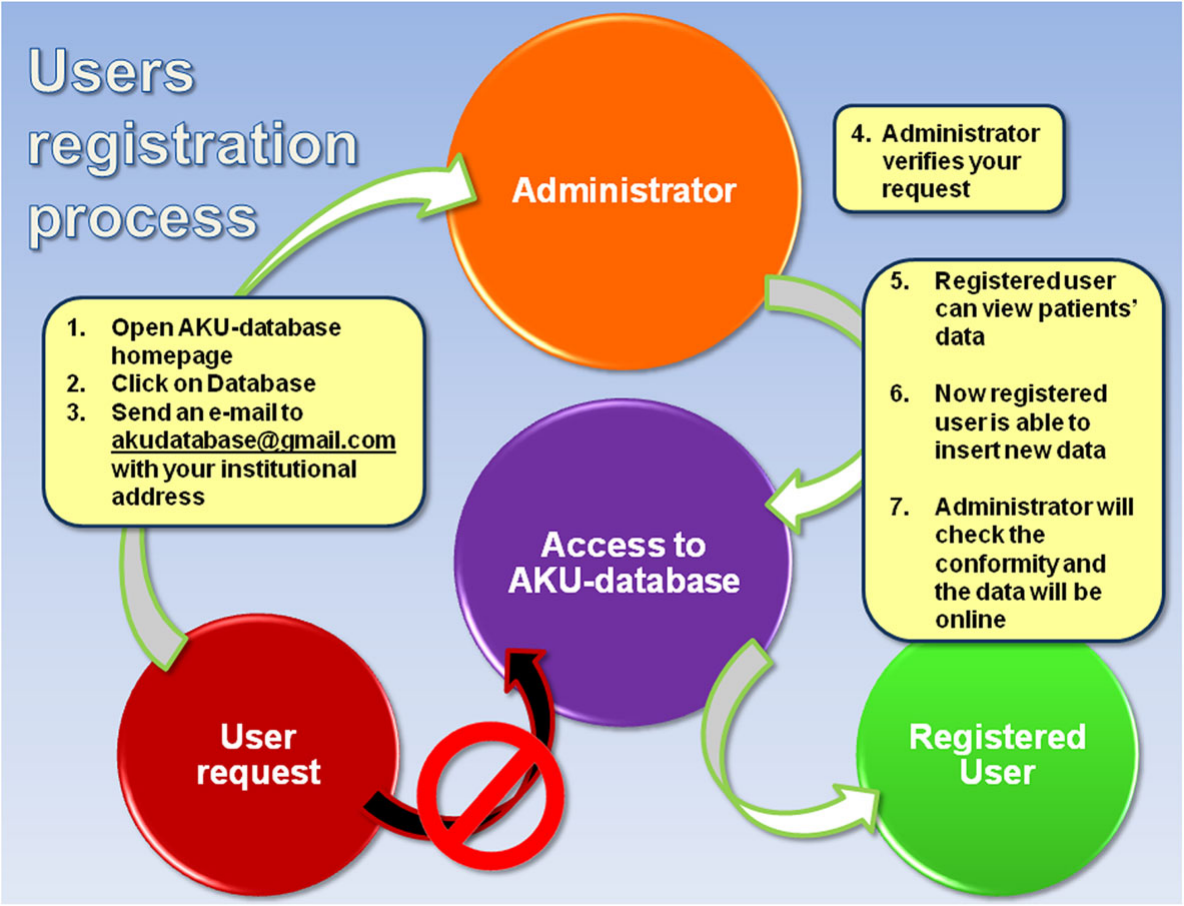
User registration process is designed so that only authorized users can be registered. When a user registers, an e-mail is automatically sent to the administrator, who has rights to confirm the user request. Once user’s registration is confirmed, he/she will receive a confirmation e-mail with username and password, with which can access the database and he/she is able to insert new data

The workflow depicted in Fig. 1 presents the registration system for for accessing the ApreciseKUre database. The system requires a registration for checking unauthorized users and for protecting information. Currently, data regarding 91 AKU patients are included in the database. In order to respect their privacy, they have been classified with a code for example: 140B or 239B where the first number indicates the gender (1 for female, 2 for male), the second and third are a progressive numbers and the last letter indicates the country of origin (A for Slovakia, B for United Kingdom, etc.). Data in the database is analyzed using classical statistical methods. The goal is to build up a dynamic model which describes how biological data are linearly correlated to each other. An algorithm has been developed to build up a refreshable correlation matrix based on Pearson’s correlation coefficient and *p-*value of each parameter and biomarker included in database. In this study, the population follows a Gaussian or normal distribution, therefore, the parametric Pearson’s statistic test has been selected. If Pearson’s coefficient *ρ* is greater than 0,3 in absolute values, the relationship between the two associated values are statistically significant. Additionally, the statistic reliability of Pearson’s index is evaluated by the *p*-value. A correlation matrix is a table containing the correlation coefficients between each variable and is used to examine the dependence between multiple variables at the same time. By visualizing every pair of variables, a complete statistical analysis can be obtained. This system gives a general picture of variable associations and its refresh feature allows the monitoring of reneweable relationships between the most recent data inserted in the database. In fact, Pearson’s index changes instantly as new datum is inserted through the appropriate database section; this change appears in the correlations matrix on the database homepage.

## Utility and discussion

**Fig. 2:**
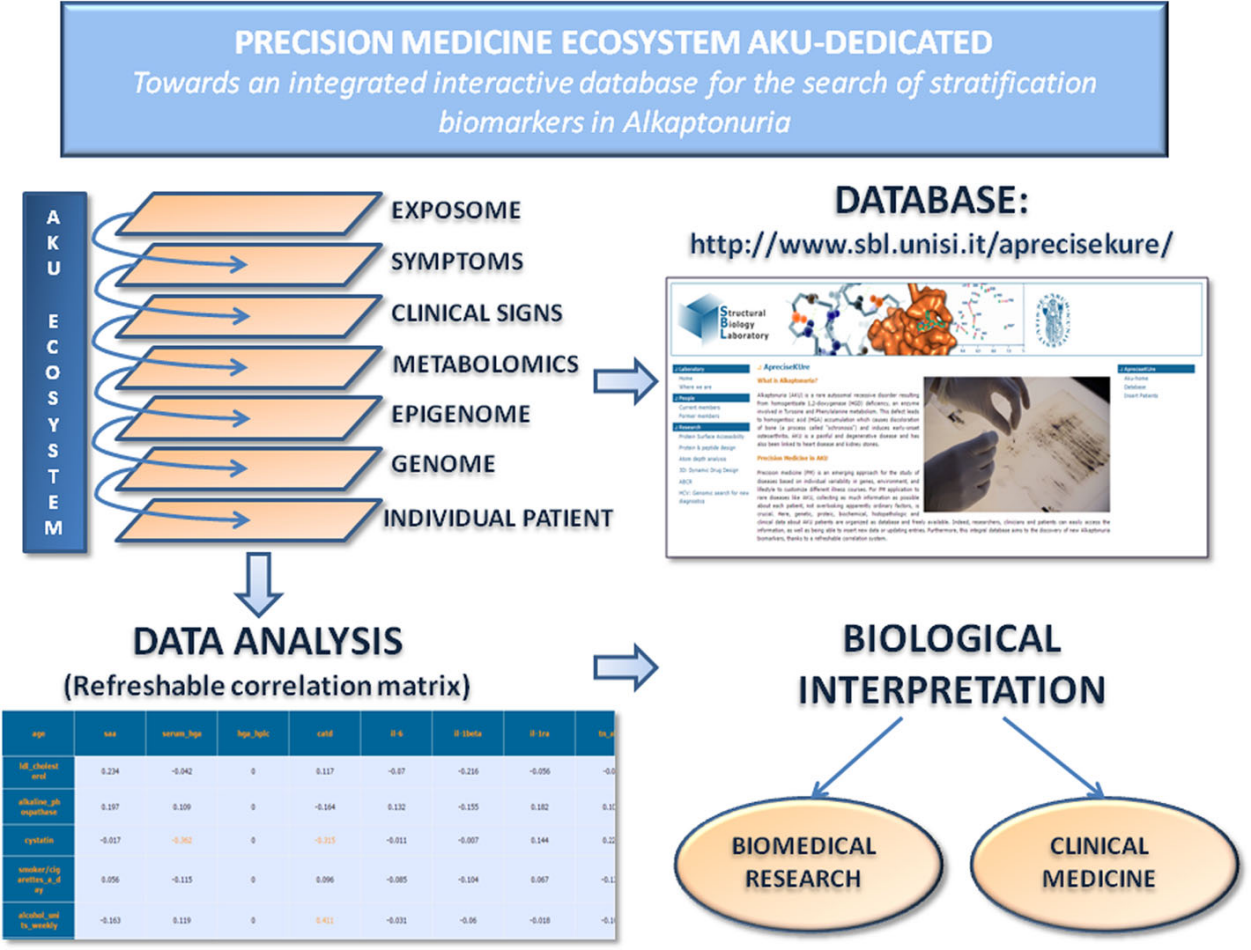
AKU-dedicated Precision Medicine Ecosystem. AKU patient data in the ApreciseKUre database are divided into various different levels: genetic, proteic, biochemical, histopathologic and clinical. Lifestyle and habitual information is also added to the total amount. Researchers, clinicians and patients could both easily access all the current information, as well as being able to insert new data, refreshing or replacing previous entries. Below you can consult Pearson’s correlations matrix, a refreshable correlation system aims to aid the discovery of AKU biomarkers. *Orange* values represent statistically significant correlations (*ρ* > 0,3). A biological interpretation is needed for a positive understanding and evaluatation of these results which could be useful not only for AKU but also offering food for thought to implement knowledge of networks between biomarkers, of other diseases and finally of biological used assays

The ApreciseKUre database aspires to become an effective tool for registered researchers, clinicians and patients who could both easily access all the current information, as well as being able to insert new data, refreshing or replacing previous entries, opening the way for an AKU-dedicated PM Ecosystem (Fig. 2). Moreover, the automatic correlation matrix gives the possibility to monitor the knowledge network underlying AKU. This feature might be useful in AKU, since patients have to combat the progressing debilitating symptoms usually for many decades, during which the therapy often needs to be adjusted individually according to the general condition of the patient.

We invite users of the ApreciseKUre database not to look at the Pearson’s index only as the numeric value, but to focus also on a deeper understanding of possible biological significance of the compaired markers. This approach can lead into discovery of interesting correlations between biomarkers not considered before and to point out eventual AKU critical points. For example, our analysis of the ApreciseKUre database indicates an inverse statistic correlation between cystatin C (CysC) and cathepsin D (CatD) (Pearson’s index −0.32 and *p*-value 0.0470).

CysC is produced at a constant rate by all nucleated cells, filtered by the renal glomeruli and catabolized in the kidney tubuli. Generally, when kidneys function properly, they stabilize the levels of CysC in the blood; if the blood levels of CysC are too high, it may indicate kidney disease [9]. CysC appears to have the advantages of being independent of gender and muscle mass, thus it is used in assays for the measurements of the glomerular filtration rate excretion (eGFR) [10, 11]. CatD is an aspartic protease synthesized as a single chain pro-enzyme (412 amino acids) in rough endoplasmic reticulum. It has been found that CatD levels are particularly elevated in muscular dystrophy and arthritis [12]. Additionally, CatD is responsible for different functions: conversion of procollagen to collagen, parathyroid hormone degradation, the splitting of some brain proteins and of CysC degradation [13].

A progressive exacerbation of ochronotic manifestations in AKU leads to kidney stones and nephrolithiasis; thus, kidney disease gradually worsens and the patient sometimes starts renal replacement therapy [14]. Interestingly, despite the fact that AKU patients often suffer from kidney dysfunction, in 40 tested patients we did not observe increased CysC levels, while CatD is elevated compared to controls (Table 1).

**Table 1:** 39 AKU patients show CatD values included between 18,76–185,2 ng/mL (measured with ELISA in serum samples) with a mean and standard deviation 62,54 ± 41,45 ng/mL

	AKU patients	Reference values
CysC mg/L (ELISA)		
Samples	29 M 11 F	---
Min-max	M:0.60–1.00 F:0.40–0.90	M:0.71–1.02^a^ F:0.58–0.98^a^
Mean ± SD	M:0.70 ± 0.15 F:0.71 ± 0.14	M:0.86 ± 0.15^a^ F:0.78 ± 0.20^a^
CatD ng/mL (ELISA)		
Samples	39	12
Min-max	18.76–185.2	36.35–56.72
Mean ± SD	62.54 ± 41.45	46.68 ± 5.88
HGA μM (ELISA)		
Samples	40	8
Min-max	13.40–54.70	0.00
Mean ± SD	25.89 ± 8.00	0.00

The outliers 18.76 ng/mL; 19.24 ng/mL and 185.2 ng/mL are responsible for the high values of standard deviation. 12 healthy control show values included between 36,35 and 56,72 ng/mL (measured with ELISA in serum samples) with a mean and standard deviation of 46,68 ± 5,88 ng/mL. 29 AKU male patients show CysC values included between 0,60 mg/L and 1,00 mg/L (measured with ELISA in serum samples) with a mean and standard deviation 0,70 ± 0,15 mg/L, 11 AKU female patients show CysC values included between 0,40 mg/L and 0,90 mg/L (measured with ELISA in serum samples) with a mean and standard deviation 0,71 ± 0,14 mg/L. Reference values differ in many populations and with sex and age: for women, the average reference interval is 0.52 to 0.90 mg/L with a mean of 0.71 mg/L. For men, the average reference interval is 0.56 to 0.98 mg/L with a mean of 0.77 mg/L [11]. 40 AKU patients show HGA values included between 13,40 and 54,70 μM (measured with ELISA in serum samples) with a mean and standard deviation 25,89 ± 8,00 μM. Thanks to correlation matrix we have verified all the possible correlations between HGA and the other biomarkers, but owing to its great variability, all the Pearson’s index show no statistically significant values

^a^[11]

This might indicate, that 1) the current version of the ApreciseKUre database concerns patients who do not show kidney dysfunction, although in all of them kidneys have to cope with excretion of high levels of HGA by both glomerular filtration and tubular secretion that leads the renal parenchyma to develop kidney stones (in 2 of them renal stones are already found) [15], or, 2) the CysC assay is not suitable for the measurements of the glomerular filtration rate excretion (eGFR) in AKU, since increased CatD can actually degrade CysC and interfere with the results.

It has been shown previously that the presence of HGA in the urine and serum interfere with standard urine creatinine enzymatic assays for kidney dysfunction [16], our present results might indicate that CysC assays is not suitable in case of AKU too, due to the high CatD. Although these results need further verification, they indicate how the proposed ApreciseKUre database can be used as an useful tool in the future for becoming aware of the failure of biomarkers clinically used and for improving the detection of more exploitable prognostic biomarkers.

## Conclusion

PM is an innovative approach that integrates research disciplines and clinical practice to build up a knowledge base that can better guide individualized patient care. In order to favor implementation of PM approach for AKU, rare inborn error of metabolism, we created a novel ApreciseKUre-database that represent suitable “PM Ecosystem” in which genetic, biochemical and clinical AKU patient’s data are shared. It is an innovative approach that gives us the potential: to investigate pathogenic mechanisms at molecular level, to define new AKU biomarkers, to undertake preclinical screening of orphan drugs, to define molecular parameters to be used as outcome measures in ongoing clinical trials and to develop a tool that accurately allows for patient stratification. Including updated case-data and samples from clinicians and patients, the researchers benefit from new information sources and also contribute to improve and increase the knowledge of the disease through data analysis. Given the chronic nature of AKU disease, it may be interesting to monitor the clinical conditions of every patient over time. The tool will be able to supply clinicians with the “weight” of each component of the complex disease (inflammation, oxidative stress, etc.), thus supporting clinical choices. Our approach can be exploited as a model of PM for other rare diseases.

## Additional file

Additional file 1: Database structure and contents. Information about database fields and stored data of collected AKU patients. (PDF 460 kb)

## Abbreviations

AKU: Alkaptonuria
BMI: Body Mass Index
CatD: Cathepsin D
ELISA: Enzyme-linked immunosorbent assay
GFR: Glomerular filtration rate
HGA: Homogentisic acid
HGD: Homogentisate 1,2-Dioxygenase
Phe: Phenylalanine
PM: Precision medicine
Tyr: Tyrosine
