# Supplementary material for: ApreciseKUre: an approach of Precision Medicine in a Rare Disease
Source: BMC Med Inform Decis Mak. 2017 Apr 14;17:42. doi: 10.1186/s12911-017-0438-0 (PMC5391600; doi:10.1186/s12911-017-0438-0)
Supplement: Additional file 1: — Database structure and contents. Information about database fields and stored data of collected AKU patients. (PDF 460 kb) [file 12911_2017_438_MOESM1_ESM.pdf]

Database structure and contents

ApreciseKUre database contains at the moment data of 91 AKU patients. The fields in the database can be grouped into 10 macroareas:

1) PATIENTS INFORMATION and MUTATIONS: including gender, birth year, country, father's and mother's origin, who is affected in family, years of diagnosis and genetic information regarding the different variants, including ID in the HGD gene mutation database.
2) OXIDATIVE STRESS, INFLAMMATION AND AMYLOIDOSIS BIOMARKERS: prognostic biomarkers are biological characteristics, which monitor the evolution of a disease and are able to distinguish severity levels outlining the natural history of each.
3) BLOOD ANALYSIS: to oversee the general health status.
4) LIFESTYLE: Data regarding patients' lifestyle, (such as cigarettes per day, alcohol units weekly, body mass index) are effective in monitoring the habits or characteristics of every patient and can provide an explanation of certain values in blood and biomarkers
5) CONCOMITANT DISEASES: AKU patients often suffer from other diseases, for instance arterial hypertension, hyperlipoproteinemia, hypothyroidism and asthma.
6) DRUGS TAKEN: especially anti-inflammatory drugs and painkillers.
7) HISTOPATHOLOGY: to assess amyloid presence in AKU specimens as cartilage, synovia, periumbelical fat and salivary glands.
8) HEALTH STATUS: a Physical and Mental Health score: self-reported measures of quality of life provide a wealth of information regarding the experience of individuals.
9) PLASMA ANALYSIS
10) URINE ANALYSIS

The data stored in the database are listed in the following table. Not every value is available in all patients:

Patients and mutations

- ID patient
- ID HGD mutation database
- Gender
- Birth year
- Year of diagnosis
- Country
- Father's origin
- Mother's origin
- Who is affected in family
- Dna change
- Protein change
- Exon/Intron
- H.10: Shannon entropy

Oxidative stress, inflammation and amyloidosis biomarkers

- SAA: Serum Amyloid A µg/mL (ELISA)
- Serum HGA: Homogentisic acid µM (ELISA)
- HGA HPLC: Homogentisic acid mmol/L (HPLC)
- CatD: Cathepsin D ng/mL (ELISA)
- IL-6: Interleukin-6 pg/mL (ELISA)
- IL-1Beta: Interleukin-1 Beta pg/mL (ELISA)
- IL-1: Interleukin Receptor antagonist pg/mL (ELISA)
- TNF alfa: Tumor necrosis factor alfa pg/mL (ELISA)
- CRP: C-reactive protein mg/dL
- CRP: C-reactive protein µg/mL (ELISA)
- MMP3: Matrix metalloproteinase 3 ng/mL (ELISA)
- AOPP: Advanced oxidation protein products µmol/dL (ELISA)

Blood analysis

- Glucose: mg/dL
- Creatinine: mg/dL
- Cholesterol: mg/dL
- Triglycerides: mg/dL
- HDL-cholesterol: mg/dL
- LDL-cholesterol: mg/dL
- Alkaline phospahatase: UI/L
- Cystatin C: mg/dL

Lifestyle

- Smoker/ cigarettes a day
- Alcohol units weekly
- BMI: Body Mass Index

Concomitant diseases and drugs taken

- Arterial hypertension
- Hyperlypoproteinemia
- Hypothyroidism
- Asthma
- Diabetes mellitus
- Hypercholesterolemia
- Ochronotic arthropathy
- Osteoporosis
- Other diseases
- Anti-inflammatories
- Painkillers
- Other drugs

Histopathology

- Congo red
- Alizarin red
- OARSI grade

Health Status

- Physical Health Score
- Mental Health Score

Plasma analysis

- HGA: Homogentisic acid mmol/L
- Tyr: Tyrosine mmol/L
- Phe: Phenylalanine mmol/L

Urine analysis

- Creatinine: mg/dL
- HGA: Homogentisic acid mg/24h
- BQA: Benzoquinone acetic acid mg/dL
